# Outcomes of Stereotactic Body Radiotherapy (SBRT) treatment of multiple synchronous and recurrent lung nodules

**DOI:** 10.1186/s13014-015-0340-9

**Published:** 2015-02-18

**Authors:** Dawn Owen, Kenneth R Olivier, Charles S Mayo, Robert C Miller, Kathryn Nelson, Heather Bauer, Paul D Brown, Sean S Park, Daniel J Ma, Yolanda I Garces

**Affiliations:** Department of Radiation Oncology, University of Michigan, 1500 E Medical Drive, Ann Arbor, MI 48105 USA; Department of Radiation Oncology, Mayo Clinic Rochester, Rochester, MN USA; Department of Radiation Oncology, MD Anderson Cancer Center, Houston, TX USA

## Abstract

**Background:**

Stereotactic body radiotherapy (SBRT) is evolving into a standard of care for unresectable lung nodules. Local control has been shown to be in excess of 90% at 3 years. However, some patients present with synchronous lung nodules in the ipsilateral or contralateral lobe or metasynchronous disease. In these cases, patients may receive multiple courses of lung SBRT or a single course for synchronous nodules. The toxicity of such treatment is currently unknown.

**Methods:**

Between 2006 and 2012, 63 subjects with 128 metasynchronous and synchronous lung nodules were treated at the Mayo Clinic with SBRT. Demographic patient data and dosimetric data regarding SBRT treatments were collected. Acute toxicity (defined as toxicity < 90 days) and late toxicity (defined as toxicity > = 90 days) were reported and graded as per standardized CTCAE 4.0 criteria. Local control, progression free survival and overall survival were also described.

**Results:**

The median age of patients treated was 73 years. Sixty five percent were primary or recurrent lung cancers with the remainder metastatic lung nodules of varying histologies. Of 63 patients, 18 had prior high dose external beam radiation to the mediastinum or chest. Dose and fractionation varied but the most common prescriptions were 48 Gy/4 fractions, 54 Gy/3 fractions, and 50 Gy/5 fractions. Only 6 patients demonstrated local recurrence. With a median follow up of 12.6 months, median SBRT specific overall survival and progression free survival were 35.7 months and 10.7 months respectively. Fifty one percent (32/63 patients) experienced acute toxicity, predominantly grade 1 and 2 fatigue. One patient developed acute grade 3 radiation pneumonitis at 75 days. Forty six percent (29/63 patients) developed late effects. Most were grade 1 dyspnea. There was one patient with grade 5 pneumonitis.

**Conclusion:**

Multiple courses of SBRT and SBRT delivery after external beam radiotherapy appear to be feasible and safe. Most toxicity was grade 1 and 2 but the risk was approximately 50% for both acute and late effects.

## Introduction

Lung SBRT has classically been used to treat solitary lung lesions in early stage non-small cell lung cancer in patients who are not candidates for primary curative resection [1,2]. Within the last 3 years, SBRT has been adopted for the treatment of oligometastatic disease in the lung from varying histologies with 2 year local control rates ranging from 75-90% [3,4]. Most series on SBRT for primary lung cancers report that the treatment of peripheral solitary lung nodules is generally well tolerated with minimal grade 3 and no grade 4/5 toxicity [3,4]. However, the impact of SBRT on the treatment of multiple lung nodules in a single treatment course or as a retreatment modality for new isolated lung nodules has not been well studied.

The low toxicity reported for lung SBRT has led to its increased use in the oligometastatic and recurrent lung cancer setting [5,6]. It is generally accepted that healthy patients with asymptomatic enlarging metastases and well controlled extrapulmonary disease can be treated with lung SBRT. One series from Indiana University analyzed their experience with treating metasynchronous and synchronous bilateral lung cancer in 10 patients. Most patients received two courses of SBRT, one to a target in each lung, over the course of 2 months. With a mean follow up of 20.7 months, no patients had more than grade 2 pulmonary toxicity [7]. A recent series of 15 patients (76 SBRT treated lesions among them) suggested that those with synchronous disease did poorly while those with metasynchronous multiple pulmonary nodules had good 2 year overall survival (68%) [8]. Once again, no patients experienced greater than grade 2 pulmonary toxicity.

As the clinical use of SBRT expands and its role in reirradiation increases, it remains unclear how many courses of lung SBRT are safe, what constitutes a safe treatment interval, and what factors predict toxicity. Further, as SBRT is widely used in asymptomatic patients with progressive lung metastases/recurrent disease, it is important to delineate who will benefit as the potential for harm is great with such large hypofractionated doses of radiation.

The current study reports on the Mayo Clinic experience with multiple courses of lung SBRT for synchronous lung nodules and treatment of metasynchronous isolated lung nodules.

## Methods

The Mayo Clinic has prospectively assessed, treated, and followed 402 patients from January 1, 2008 to August 1, 2012 with SBRT for lung nodules. Of these, 63 patients received multiple courses of lung SBRT or SBRT following high dose external beam radiotherapy (EBRT) to the mediastinum for 128 sites treated. Information collected on these patients included: patient age, gender, tumour histology, number of metastases at simulation, number of nodules treated, site of lung nodules, synchronous and metasynchronous treatment, local control, distant progression, radiographic response to treatment, SBRT prescription dose, chemotherapy delivery, acute and late toxicity. Descriptive statistics were performed using the in house Mayo clinic program JMP (Version 9.01, SAS Institute Inc., Cary, North Carolina, USA).

Progression free survival, overall survival, and follow up from the end of SBRT treatment were estimated using the Kaplan Meier method. Progression free survival was defined as any local or distant progression following the end of SBRT treatment. Local failure was defined as in-field progression over serial CT-based imaging. This study was approved by the Mayo Clinic Institutional IRB ethics board.

SBRT plans were designed using Eclipse (Varian, Palo Alto, CA, USA) treatment planning software. All patients underwent 4DCT planning scans. The ITV was defined by contours on 10 phases of respiration. Expansion from ITV to PTV was generally 5 mm circumferentially in all directions. Abdominal compression and breath hold techniques were employed to minimize motion in lower lobe tumours. Prior to 2010, most patients received lung SBRT every other day and planning was performed using 3DCRT and static field IMRT techniques. After 2010, VMAT/RapidArc® planning was more frequently used and treatment was delivered on a daily basis even in those with previous lung SBRT treatments. Based on our experience and other published reports, we have found no treatment planning effect on local control or difference in toxicity among all lung SBRT patients [9,10]. Daily cone beam CT was used to verify the position of the ITV prior to each treatment delivery.

A common set of dose volume histogram (DVH) metrics were calculated for all patients in the study using an in-house program, DataMiner. Structures contoured for DVH analysis included the heart, esophagus, bilateral lungs, great vessels, spinal cord, trachea, and proximal bronchial tree as definied by RTOG 0236. Dependencies of toxicity scores on DVH metrics were examined using Receiver Operator Characteristics (ROC) curve analysis, carried out with R (The R Foundation for Statistical Computing, www.r-project.org). The area under the ROC curves (ROC AUC) was calculated. For each DVH metric, a threshold value was determined as the value maximizing the Youden Index on the ROC curve. The significance of the association of groups segregated into 2x2 contingency tables according to toxicity and value of each DVH metric with respect the threshold was calculated using Fisher’s exact test.

While data was available in a prospectively collected Mayo Clinic database, all data was verified by retrospective chart review. Acute and late toxicity data were documented at every follow up in a prospective manner. Additional information was gleaned from follow up notes and notes documenting effects during the treatment course. The standardized CTCAE version 4.03 scale was applied retrospectively to these documented effects.

## Results

### Patient demographics

A total of 128 lung nodules were treated in 63 patients with lung SBRT during the study period (Table 1). Of the 63 patients, 18 had received prior chest/mediastinum radiotherapy before receiving SBRT (median EQD2 EBRT dose received was 60 Gy). More than half of the patients were female (34/63) and more than half of the tumours treated were primary lung cancers (41/63). Mean tumour size was 1.8 cm (range 0.6 to 5.0 cm). Approximately 1/3 of the tumours were centrally located (47/128). The most common prescription dose was 50 Gy/5 fractions and 54 Gy/3 fractions. Most patients had 2 lung nodules (42/63) treated. Of these, 28 patients had two lung sites treated with consecutive courses of SBRT separated by one month. The median time between lung SBRT courses for all patients was 12.9 months (0–113 months). The median time beween lung SBRT and prior EBRT was 18.4 months (range 1.5-112 months).

**Table 1:** **Demographics of multiple courses lung SBRT patients**

**Gender**	**N=63**
Male	29
Female	34
**Median age at tx**	72 (range 20-90)
**Histology**	**N=63**
Breast cancer	1
Head & neck	5
Non small cell lung cancer	40
Small cell lung cancer	1
Melanoma	4
Renal cell carcinoma	2
Skin cancer	1
Colon ca	6
Thyroid	1
Sarcoma	2
**Prior definitive lung external beam radiotherapy**	**N=63**
Yes	18
No	45
**Central vs peripheral lesion**	**N=128**
Central	47
Peripheral	81
**Mean tumour diameter (longest dimension)**	**N=128**
	1.8 cm (range 0.6 to 5.0 cm)
**Primary v recurrent v metastatic disease**	**N=128**
Primary lung tumour	40
Recurrence after primary SBRT for T1N0 disease	25
Metastatic lung nodules	45
Recurrence after EBRT for locally advanced lung cancer	15
Unknown	3
**Prescription dose**	**N=128**
40 Gy/5#	3
45 Gy/9#	1
45 Gy/5#	1
48 Gy/4#	30
50 Gy/10#	2
50 Gy/5#	43
54 Gy/3#	43
57.5 Gy/5#	1
60 Gy/3#	5
**Number of sites treated per patient**	**N=63**
1	12
2	42
3	6
4	3
**Number of SBRT courses per patient**	**N=63**
1 (synchronous; 2 sites treated at once)	29
2	28
3	6
**Response to treatment (based on imaging)**	**N=128**
CR	21
PR	63
Progression	3
SD	26
Unknown	16
**Chemo given**	**N=63**
Yes	17
No	46

### Dosimetry

Cumulative lung dose was calculated using sum plans within the Varian® Eclipse planning software. The cumulative mean lung volume irradiated in all patients was 3363 cc with a mean DC1000cc of 2.9 Gy and DC1500cc of 8.2 Gy. Mean lung V5 was 43% (median 39%) and mean V20 was 15% (median 12%). Mean lung dose was 10.1 Gy (median 8.5 Gy). The mean BED received by both lungs across cumulative SBRT courses was 255 Gy. Mean heart dose was 9.5 Gy (median 6.0 Gy). Cumulative maximum dose to the chest wall ranged from 25–142 Gy (median 66 Gy) with a mean D5cc of 58.6 Gy. Cumulative maximum esophageal dose ranged from 7.1 to 79.4 Gy (median 24.5 Gy). Cumulative D4cc bronchial tree threshold dose ranged from 0.2-79.6 Gy (median 17.6 Gy).

### Acute toxicity

Acute toxicity was any toxicity related to lung SBRT occurring < 90 days from the start of SBRT (Table 2). 51% of patients (32/63 patients) experienced acute toxicity with the majority (44 events) grade 2 or less. Half of the patients with acute toxicity were those who had synchronous lung SBRT courses or consecutive SBRT courses separated by one month. 40% of the patients who experienced toxicity had prior lung EBRT; however, none of these patients experienced greater than grade 2 toxicity. The most common toxicity was fatigue (16/44). The median time to acute toxicity was 25 days (range 1–89 days). There were two grade 3 toxicities: one was radiation pneumonitis requiring hospitalization and the second was hepatitis requiring hospitalization. The latter was questionable as the right lower lobe lesion treated in this case was approximately 2 cm from the liver edge.

**Table 2:** **Acute toxicity (N = 44 events; N = 32 patients)**

**Median time to acute toxicity**	**25 days (range 1-89 days)**
**Prescription dose**	**N=32**
40 Gy/5#	2
45 Gy/5#	1
48 Gy/4#	7
50 Gy/10#	2
50 Gy/5#	9
54 Gy/3#	7
60 Gy/3#	3
57.5 Gy/5#	1
**Prior definitive EBRT**	**N=32**
Yes	9
No	23
**Grade toxicity**	**N=44**
1	31
2	11
3	2
**Type of toxicity**	**N=44**
Chest wall pain	8
Cough	4
Dermatitis	2
Dyspnea	5
Fatigue	16
Hemoptysis	1
Hepatitis (questionable)	1
Hoarseness	2
Nausea	3
Odynophagia	1
Pneumonitis (at 75 days)	1
**Number of SBRT courses per patient**	**N=32**
1	12
2	16
3	4
**Number of sites treated per patient with SBRT**	**N=32**
1	4
2	21
3	4
4	3

On univariate analysis, chemotherapy delivered within 1 month of SBRT (p = 0.03), prior lung EBRT (p = 0.01), left sided lesions (p = 0.001), and higher BED or EQD2 (p = 0.01), were associated with developing acute toxicity. These three factors were not associated with the development of acute toxicity on multivariate analysis. No dosimetric variables were correlated with acute toxicity.

### Late toxicity

Median followup from the end of SBRT was 12.6 months (range 0.5 to 50 months). Nearly half of the patients (29/63) had some late effects (toxicity arising beyond 90 days from the end of SBRT Table 3). The majority were less than grade 2 (33/37). Of the 29 patients with late toxicity, 8 had prior chest EBRT. The most common late effect was dyspnea. The median time to late toxicity was 192 days (range 91 to 483 days). Of the three patients with grade 3 toxicities, two developed severe dyspnea requiring hospitalization and continuous oxygen therapy afterwards. The third grade 3 toxicity was left vocal cord paralysis after treatment of a left upper lobe lesion requiring injection of the affected vocal cord. There was one grade 5 toxicity where a patient died from radiation pneumonitis (162 days after his last course of SBRT). None of the patients who developed grade 3 or higher late toxicity had received prior EBRT to the chest. This patient had a single right lung nodule radiated to 50 Gy/5 fractions SBRT and six months later received a course of lung SBRT to two synchronous nodules (one in the right upper lobe and another in the left lower lobe). His first course of SBRT was to a central lesion and the second course was to one central and one peripheral lesion.

**Table 3:** **Demographics of late effects (N = 37 events; N = 29 patients)**

**Median time to late toxicity**	**192 days (range 91-483 days)**
**Primary v recurrent v metastatic disease**	**N=29**
Primary	11
Recurrent	12
Metastatic	6
**Prescription dose**	**N=29**
45 Gy/5#	1
48 Gy/4#	5
50 Gy/1o0#	1
50 Gy/5#	9
54 Gy/3#	10
60 Gy/3#	3
**Prior definitive EBRT**	**N=29**
Yes	8
No	21
**Grade toxicity**	**N=37**
1	18
2	15
3	3
5	1 (one patient died of pneumonitis at d162)
**Type of toxicity**	**N=37**
Chest wall pain	6
Lymphedema	1
Cough	6
Dyspnea	13
Vocal cord paralysis	1
Fatigue	2
Pneumonitis (>90 days)	5
Hemoptysis	1
Necrosis	1
Rib fracture	1
**Number of courses SBRT per patient**	**N=29**
1	10
2	13
3	6
**Number of sites treated per patient**	**N=29**
2	18
3	8
4	3
**Number who had 2 sites treated at once/split over short period of time between 2 courses**	**N=12**

On univariate analysis, gender (male; p = 0.01), lung histology (p = 0.03) and number of SBRT sites treated (p = 0.0001) were associated with late toxicity. None of these factors were predictive of late toxicity on multivariate analysis. None of the lung or heart dosimetric parameters were correlated or predictive of late toxicity.

### Local control

Of the 128 sites treated with multiple courses of SBRT, 6/128 showed local recurrence or progression and 110/128 had stable disease or better. More than half of the treatment courses, 80/128 had distant progression after the end of SBRT. Actuarial local control at 2 years was 99.1%. Given the very low failure rate, it was not possible to examine any factors that predicted for local failure.

### Overall survival and progression free survival

Median SBRT overall survival (OS) was 35.7 months (0.6 to 51.0 months) and median SBRT progression free survival (PFS) was 10.7 months (0.5 to 55.8 months). One year overall survival from the end of SBRT treatment was 85% while one year progression free survival was 91.9%. Histology (primary lung cancer versus metastatic disease) had no impact on SBRT specific overall survival. However, SBRT PFS was much higher in patients with lung cancer than those with oligometastatic disease (p = 0.0001; median lung cancer SBRT PFS was 15.4 months versus 3.8 months in oligometastatic lung disease and 12 month SBRT PFS 62.0% in lung patients versus 10.7% in oligometastatic disease).

## Discussion

The current study is one of the largest institutional reviews of repeated courses of lung SBRT for primary lung cancers and lung oligometastases. In our study, the local control rate was 99.1% at 24 months. The low risk of local recurrence is reflective of the high BED of SBRT treatment. The largest published series to date on SBRT treatment of multiple primary lung cancers (74 lesions treated among 48 patients) noted a crude local control rate of 93% (only 6 patients failed) with a median follow up of 24 months [8]. Previous reviews have demonstrated similar results but with much smaller numbers (10 patients). One report from the University of Oklahoma treated 10 patients with 21 synchronous/metachronous inoperable lung tumours with an 18 month local control rate of 95% [11]. The University of Indiana also published a series on 10 patients with an 18 month local control rate of 80% for patients with bilateral multiple biopsy proven non small cell lung cancers [7].

In the oligometastatic setting, little has been published on multiple courses of SBRT. The largest study to date is from China and examined 172 lung lesions in 71 patients treated with SBRT. With a median follow up of 24 months, they noted local control rates of 75% at 2 years [12]. Another study reporting on 61 patients treated primarily for solitary lung metastases (74% of patients in this series) of various histologies demonstrated a 2 year local control rate of 89% [3]. A handful of series have also compared the local control rates for oligometastatic disease (predominantly colorectal cancer) and primary lung cancer treated with SBRT. They demonstrated a statistically worse local control rate for oligometastatic disease (78-82% at 2 years) than primary lung cancer treated with SBRT (93-98% at 2 years) [4,13,14]. Colorectal cancer has historically been shown to have worse local control than breast metastases [15,16]. Given our very low failure rate (only 46 sites failed out of 128), it was not possible to explore factors that predicted for worse local control.

Our patient population had an excellent median overall survival. Primary lung cancer histology was correlated with much improved progression free survival in our series. This is not surprising as those treated for primary lung cancers generally had disease limited to the lungs while oligometastatic patients may have had other systemic disease that was quiescent at the time of treatment. In the primary lung cancer setting, it has been suggested that synchronous tumours do worse than metachronous tumours treated with SBRT as synchronous tumours at presentation reflect a higher initial disease burden. Creach et al. noted superior 2 year overall survival (53% versus 0%) and progression free survival (68% versus 27%) in SBRT lung patients treated for metachronous versus synchronous disease [8]. We did not find any interaction of synchronous versus metachronous tumours on survival in primary lung tumour patients or those with oligometastatic disease. In the oligometastatic setting, 2 year overall survival rates of 57- 66% and progression free survival rates of 32.4% have been reported [3,13]. Our findings are comparable to these series.

In our patient cohort, there was a 50% chance of developing early toxicity (defined as ≤ 90 days from the last day of SBRT treatment). Most of these were grade 1 toxicities. In our series, fatigue was the most common symptom although one patient did develop grade 3 radiation pneumonitis at 75 days. This is consistent with previous reports on SBRT for primary lung tumours where grade 1 fatigue was noted in approximately half of treated patients [17].

Much of the lung SBRT literature has focused on the risk factors for radiation pneumonitis. We examined risk factors for all grade 2 and above pulmonary toxicities (dyspnea, cough, and radiation pneumonitis). In the current study, we did not find any factor predictive of late pulmonary toxicity. Prior studies have shown that central lesions retreated with SBRT may be at increased risk of toxicity. Peulen et al. reported 3 deaths from grade 5 hemorrhage secondary to reirradiation with SBRT of centrally located lesions although in these cases, the former PTV overlapped the subsequent PTV by ≥ 50% [18]. Other studies in which individual patients likely received more than one course of lung SBRT showed minimal toxicity with no grade 3 acute or late toxicities [8,12]. One patient in our series succumbed to respiratory distress at 162 days. It was equivocal whether this was directly related to his SBRT treatment as he also had severe underlying COPD but he was still coded as a potential grade 5 toxicity. Many groups advocate a risk adapted strategy for central lesions with demonstration of similar SBRT toxicity profiles between peripheral and central lesions if central lesions are treated to a lower BED over a more prolonged fractionated course [19-22]. At the Mayo Clinic, central lesions typically are treated with a dose of 48 Gy/4 fractions or 50 Gy/5 fractions and peripheral lesions receive 54 Gy/3 fractions. The safest dose for central lesions is still the subject of an ongoing RTOG trial (0813).

In the current study, we did not find that any lung dosimetric parameters that were correlated with the development of any grade 2 or above pulmonary toxicity. It was difficult to find any correlation given the low risk of severe complications. We tried to group complications together to see if we could find any hypothesis generating associations. The lack of connection between DVH analysis and lung toxicity is not surprising given the clinical equipoise surrounding what lung parameters are the most salient. Multivariate analyses from a number of institutions have yielded heterogeneous and non-congruent dosimetric predictors. The most commonly studied dosimetric measurements are mean lung dose, V5, V20, and contralateral lung dose [23,24]. There is also some evidence that centrally located lesions lead to higher mean lung doses by virtue of trying to push a higher dose through more lung [25]. However, other studies allude to the possibility that patient factors such as underlying COPD may be more salient or more esoteric lung parameters such as V25 may be important [26,27]. In the end, it is often difficult to ascribe late pulmonary symptoms to SBRT treatment in the setting of naturally evolving and progressive COPD.

Most recently, heart dose has emerged as a cause for concern in lung SBRT. The mechanism of how this relates to the risk of radiation pneumonitis is being elucidated. We did not observe this correlation in our patient cohort although, interestingly, when patients with prior lung EBRT were excluded, the mean heart dose was correlated with a higher risk of grade 3 or above pulmonary toxicity. In SBRT planning, we seldom place mean organ dose constraints and are more likely to limit organs to a volumetric threshold dose or D_max._ To date, few SBRT studies have examined the impact of heart dose as a predictor for radiation pneumonitis. In the setting of locally advanced lung cancer treated with radical chemoradiotherapy, some groups have broached the possibility that heart irradiation is correlated with the development of radiation pneumonitis. Huang et al. examined the heart and lung dose volume parameters for 209 patients and found that the most significant variables associated with ≥ grade 2 radiation pneumonitis were heart V65 and heart D10 [28]. Lung dosimetric variables minimally changed the modeling for the risk of radiation pneumonitis implying that the heart dose is not simply a surrogate for lung dose. A Chinese study confirmed these findings showing the mean heart dose and DVH-CV was correlated with the risk of grade ≥ 2 radiation pneumonitis. However, this was only significant on univariate analysis and was no longer predictive on multivariate logistic regression [29]. Animal studies from the Groningen group have shown that radiation of the heart compromises lung function although the exact mechanism has yet to be elucidated [30,31].

The current study shows that multiple courses of lung SBRT is feasible and that the risk of severe (grade ≥ 2) pulmonary toxicity is uncommon. However, there are several limitations including the retrospective nature of data verification (although patients were entered into a prospective database), the lack of patient reported outcomes, and short follow up for greater delineation of late effects. Patient factors such as baseline pulmonary function, severity of COPD prior to SBRT, cardiac comorbidities, and ongoing smoking history were also not consistently documented. These may also have an impact on the risk of acute and late effects as well.

Generally, multiple courses of SBRT either for synchronous lung lesions or metachronous lung lesions are very tolerable. None of the patients who had received prior lung EBRT developed higher than grade 2 toxicity. Most toxicity in the acute and late setting were grade 1 with fatigue and dyspnea being the most common symptoms. Local control was excellent (in excess of 90% at 2 years) with excellent median overall survival. The emergence of heart dose as a possible predictor of radiation pneumonitis is interesting and warrants more investigation as reirradiation of centrally located lung tumours with SBRT may require development of further risk adapted strategies.

## References

[1] Timmerman R, Abdulrahman R, Kavanagh BD, Meyer JL (2007). Lung cancer: a model for implementing stereotactic body radiation therapy into practice. Front Radiat Ther Oncol.

[2] Timmerman RD, Park C, Kavanagh BD (2007). The North American experience with stereotactic body radiation therapy in non-small cell lung cancer. J Thorac Oncol.

[3] Ricardi U, Filippi AR, Guarneri A, Ragona R, Mantovani C, Giglioli F (2012). Stereotactic body radiation therapy for lung metastases. Lung Cancer.

[4] Takeda A, Kunieda E, Ohashi T, Aoki Y, Koike N, Takeda T (2011). Stereotactic body radiotherapy (SBRT) for oligometastatic lung tumors from colorectal cancer and other primary cancers in comparison with primary lung cancer. Radiother Oncol.

[5] Pan H, Rose BS, Simpson DR, Mell LK, Mundt AJ, Lawson JD (2013). Clinical practice patterns of lung stereotactic body radiation therapy in the United States: a secondary analysis. Am J Clin Oncol.

[6] Amini A, Yeh N, Gaspar LE, Kavanagh B, Karam SD (2014). Stereotactic body radiation therapy (SBRT) for lung cancer patients previously treated with conventional radiotherapy: a review. Radiat Oncol.

[7] Sinha B, McGarry RC (2006). Stereotactic body radiotherapy for bilateral primary lung cancers: the Indiana University experience. Int J Radiat Oncol Biol Phys.

[8] Creach KM, Bradley JD, Mahasittiwat P, Robinson CG (2012). Stereotactic body radiation therapy in the treatment of multiple primary lung cancers. Radiother Oncol.

[9] Stauder MC, Macdonald OK, Olivier KR, Call JA, Lafata K, Mayo CS (2011). Early pulmonary toxicity following lung stereotactic body radiation therapy delivered in consecutive daily fractions. Radiother Oncol.

[10] Yamashita H, Haga A, Takahashi W, Takenaka R, Imae T, Takenaka S (2014). Volumetric modulated arc therapy for lung stereotactic radiation therapy can achieve high local control rates. Radiat Oncol.

[11] Matthiesen C, Thompson JS, De La Fuente HT, Ahmad S, Herman T (2012). Use of stereotactic body radiation therapy for medically inoperable multiple primary lung cancer. J Med Imaging Radiat Oncol.

[12] Zhang Y, Xiao JP, Zhang HZ, Yin WB, Hu YM, Song YX (2011). Stereotactic body radiation therapy favors long-term overall survival in patients with lung metastases: five-year experience of a single-institution. Chin Med J (Engl).

[13] Oh D, Ahn YC, Seo JM, Shin EH, Park HC, Lim do H (2012). Potentially curative stereotactic body radiation therapy (SBRT) for single or oligometastasis to the lung. Acta Oncol.

[14] Hamamoto Y, Kataoka M, Yamashita M, Nogami N, Sugawara Y, Kozuki T (2012). Factors affecting the local control of stereotactic body radiotherapy for lung tumors including primary lung cancer and metastatic lung tumors. Jpn J Radiol.

[15] Takeda A, Kunieda E, Sanuki N, Aoki Y, Oku Y, Handa H (2012). Stereotactic body radiotherapy (SBRT) for solitary pulmonary nodules clinically diagnosed as lung cancer with no pathological confirmation: Comparison with non-small-cell lung cancer. Lung Cancer.

[16] Milano MT, Katz AW, Zhang H, Okunieff P (2012). Oligometastases treated with stereotactic body radiotherapy: long-term follow-up of prospective study. Int J Radiat Oncol Biol Phys.

[17] Taremi M, Hope A, Dahele M, Pearson S, Fung S, Purdie T (2012). Stereotactic body radiotherapy for medically inoperable lung cancer: prospective, single-center study of 108 consecutive patients. Int J Radiat Oncol Biol Phys.

[18] Peulen H, Karlsson K, Lindberg K, Tullgren O, Baumann P, Lax I (2011). Toxicity after reirradiation of pulmonary tumours with stereotactic body radiotherapy. Radiother Oncol.

[19] Bral S, Gevaert T, Linthout N, Versmessen H, Collen C, Engels B (2011). Prospective, risk-adapted strategy of stereotactic body radiotherapy for early-stage non-small-cell lung cancer: results of a Phase II trial. Int J Radiat Oncol Biol Phys.

[20] Chang JY, Balter PA, Dong L, Yang Q, Liao Z, Jeter M (2008). Stereotactic body radiation therapy in centrally and superiorly located stage I or isolated recurrent non-small-cell lung cancer. Int J Radiat Oncol Biol Phys.

[21] Chi A, Liao Z, Nguyen NP, Xu J, Stea B, Komaki R (2010). Systemic review of the patterns of failure following stereotactic body radiation therapy in early-stage non-small-cell lung cancer: clinical implications. Radiother Oncol.

[22] Fakiris AJ, McGarry RC, Yiannoutsos CT, Papiez L, Williams M, Henderson MA (2009). Stereotactic body radiation therapy for early-stage non-small-cell lung carcinoma: four-year results of a prospective phase II study. Int J Radiat Oncol Biol Phys.

[23] Grimm J, Palma D, Xue J, Senan S (2012). SU-E-T-246: Preliminary Normal Tissue Complication Probability (NTCP) Analysis for Radiation Pneumonitis (RP) after Stereotactic Body Radiotherapy (SBRT). Med Phys.

[24] Barriger RB, Forquer JA, Brabham JG, Andolino DL, Shapiro RH, Henderson MA (2012). A dose-volume analysis of radiation pneumonitis in non-small cell lung cancer patients treated with stereotactic body radiation therapy. Int J Radiat Oncol Biol Phys.

[25] De La Fuente HT, Vlachaki MT, Herman TS, Hibbitts K, Stoner JA, Ahmad S (2010). Stereotactic body radiation therapy (SBRT) and respiratory gating in lung cancer: dosimetric and radiobiological considerations. J Appl Clin Med Phys.

[26] Takeda A, Ohashi T, Kunieda E, Sanuki N, Enomoto T, Takeda T (2012). Comparison of clinical, tumour-related and dosimetric factors in grade 0–1, grade 2 and grade 3 radiation pneumonitis after stereotactic body radiotherapy for lung tumours. Br J Radiol.

[27] Matsuo Y, Shibuya K, Nakamura M, Narabayashi M, Sakanaka K, Ueki N (2012). Dose-volume metrics associated with radiation pneumonitis after stereotactic body radiation therapy for lung cancer. Int J Radiat Oncol Biol Phys.

[28] Huang EX, Hope AJ, Lindsay PE, Trovo M, El Naqa I, Deasy JO (2011). Heart irradiation as a risk factor for radiation pneumonitis. Acta Oncol.

[29] Dang J, Li G, Ma L, Diao R, Zang S, Han C (2013). Predictors of grade >/= 2 and grade >/= 3 radiation pneumonitis in patients with locally advanced non-small cell lung cancer treated with three-dimensional conformal radiotherapy. Acta Oncol.

[30] van Luijk P, Faber H, Meertens H, Schippers JM, Langendijk JA, Brandenburg S (2007). The impact of heart irradiation on dose-volume effects in the rat lung. Int J Radiat Oncol Biol Phys.

[31] van Luijk P, Novakova-Jiresova A, Faber H, Schippers JM, Kampinga HH, Meertens H (2005). Radiation damage to the heart enhances early radiation-induced lung function loss. Cancer Res.

